# Effect of steroid treatment on the diagnostic yield of baseline ^18^f-fluorodeoxyglucose positron emission tomography in aggressive B cell lymphoma

**DOI:** 10.1186/s13550-022-00924-9

**Published:** 2022-09-14

**Authors:** Karyn Revital Geiger, Oren Pasvolsky, Tamar Berger, Pia Raanani, Tzippy Shochat, Ronit Gurion, Tamer Anati, David Groshar, Anat Gafter-Gvili, Hanna Bernstine

**Affiliations:** 1 Davidoff Cancer Center, Institute of Hematology, Rabin Medical Center – Beilinson Hospital, 4941492 Petach Tikva, Israel; 2 Sackler Faculty of Medicine, Tel Aviv University, Tel Aviv, Israel; 3 Biostatistical Unit, Rabin Medical Center – Beilinson Hospital, Petach Tikva, Israel; 4 Department of Medicine A, Rabin Medical Center – Beilinson Hospital, Petach Tikva, Israel; 5 Department of Nuclear Medicine, Rabin Medical Center – Beilinson Hospital, Petach Tikva, Israel

**Keywords:** Lymphoma, PET CT, Steroid

## Abstract

Aggressive B cell lymphoma often requires prompt steroid treatment, even before baseline ^18^f-fluorodeoxyglucose (FDG) positron emission tomography/computed tomography (PET/CT) and definitive treatment, to alleviate symptoms or prevent organ damage. Since lymphoma is a steroid-sensitive malignancy, there are concerns that steroids might affect the results of FDG PET/CT and decrease its diagnostic yield. The aim of the current study was to evaluate the effect of steroids administered before baseline PET/CT on the maximum standardized uptake value (SUVmax) and additional PET/CT parameters. Retrospective review of the database in a tertiary medical center yielded 178 patients newly diagnosed with aggressive B cell lymphoma between January 2017 and May 2020 who had an available baseline FDG PET/CT scan. The cohort was divided into patients who received steroids before PET/CT (*n* = 47) and those who did not (*n* = 131), and the groups were compared for SUVmax and additional PET/CT parameters. The steroid-treated group had a higher disease stage and lactate dehydrogenase level compared to the steroid-naïve group, with a trend toward a higher international prognostic index. There was no significant between-group difference in SUVmax (*P* = 0.61). This finding remained consistent across steroid treatment durations and dosage regimens. Further evaluation revealed a significantly larger mean tumor volume and a trend toward a higher tumor metabolic burden in the steroid-treated group, yet no between-group difference in SUV mean or other PET/CT parameters. In this retrospective analysis of patients with aggressive B cell lymphoma, steroid prophase prior to baseline PET/CT did not decrease the diagnostic yield of the scan. However, further studies are required to fully appreciate the impact of steroids on PET CT parameters.

## Introduction

^18^F-fluorodeoxyglucose (FDG) positron emission tomography/computed tomography (PET/CT) is a functional imaging technique that reflects the metabolic rate of tissues and has been used to measure the increased metabolic demand in tumor cells. It currently serves as a mainstay of malignancy surveillance in high-grade lymphomas and has been incorporated into treatment guidelines. Its use was found to significantly improve the accuracy of initial staging and monitoring of the treatment response [1–3].

The uptake of FDG in tumors, however, may be affected by blood glucose levels. There are conflicting data regarding the effect of glucose on FDG uptake in tumors. Several reports have found blood glucose levels to have a minor impact on FDG uptake in malignancy, mostly restricted to profound hyperglycemia or the specific uptake of brain tumors [4–7]. Steroids are part of the treatment protocols of aggressive lymphomas, such as rituximab, cyclophosphamide, doxorubicin, vincristine, and prednisone (R-CHOP) and hyperglycemia is one of their well-documented side effects [8, 9]. They are often initiated empirically after biopsy, before the final histopathological diagnosis and staging, in order to alleviate clinical symptoms or prevent impending organ damage.

There are several reports in the literature describing disruptive effects of steroid treatment on PET/CT results, mostly in patients with rheumatological diseases, such as giant cell arteritis. The possible impact of steroids might be linked to duration and dosage of steroid treatment prior to PET CT scan [10, 11]. Yet, there is paucity of data regarding the possible effect of steroids on PET/CT findings in lymphoma patients, and there is concern that initiating steroid treatment before the baseline PET/CT scan might affect interpretation of the scan.

The aim of the present study was to evaluate the impact of steroid treatment prior to PET/CT examination on the maximum standardized uptake value (SUVmax) and other baseline PET/CT parameters in patients with newly diagnosed aggressive B cell lymphoma.

## Methods

### Study design, setting and participants

The electronic healthcare database of the hemato-oncology unit of a tertiary cancer center was retrospectively reviewed for all consecutive adult patients (age ≥ 18 years) with newly diagnosed aggressive non-Hodgkin's B cell lymphoma between January 2017 and May 2020. We included patients with diffuse large B cell lymphoma, high-grade B cell lymphoma, primary mediastinal (thymic) large B cell lymphoma, and Burkitt lymphoma, all of which are associated with symptoms and a risk of organ damage and therefore often warrant prompt steroid treatment. We included patients who underwent PET/CT examination as part of the routine clinical workup. Patients were divided into those who received steroid treatment (any dose, any route) within 30 days before the PET/CT scan, and those who did not.

The study was approved by the Institutional Review Board of Rabin Medical Center.

### Data collection

Clinical data were collected from the medical records, as follows: demographics, histologic diagnosis according the WHO 2016, lymphoma characteristics, steroid treatment (if any) and dose, glucose level up to 24 h prior to PET CT in milligram (mg)/deciliter (dL) and date of performance of PET/CT. The lymphoma characteristics included lactate dehydrogenase (LDH) levels (upper limit of normal in our institution, 480 U/L), ki67 proliferation index, disease stage (according to the Lugano classification), and international prognostic index (IPI) [12]. The different steroid formulations (dexamethasone, hydrocortisone etc.) were converted to prednisone equivalent doses using a standard conversion equation [13, 14].

### PET/CT scan acquisition

PET CT scans were performed in various PET CT facilities in Israel (mainly in our institution), and all PET CTs were reviewed in our institution by physicians who specialize in nuclear medicine.

SUV is a semiquantitative index calculated by the ratio of FDG concentration in a selected region of interest to the injected dose which is normalized to body weight. SUVmax reflects the maximal value within a selected volume of interest; SUVmean reflects the average value in the volume of interest.

Metabolic tumor volume (MTV) is a measure of the volume of the metabolically active areas of the tumor in the volume of interest, calculated by threshold from the SUVmax. Total lesion glycolysis (TLG) is the product of SUVmean and MTV.

Segmentation was performed using CARESTREAM-PACS software, version 12.1.5.7014, which automatically defines the contour of the PET-based lesion. The cut-off is 42% of the tumoral SUVmax.

Total metabolic burden was derived from the TLG (SUVmean* MTV), with the volume of interest of the whole scan and reduction of physiological FDG uptake in the brain, heart, kidneys and bladder. The tumor volume was defined as the MTV calculated in this volume of interest.

We also calculated the SUVmax and SUVmean of the liver (3 cm diameter spherical volume of interest, segment 7) and mediastinum (1.5 cm diameter spherical volume of interest, placed on the aortic arch) and the lesion-to-liver SUVmax ratio.

### Study procedure

Patients who received steroids were compared to steroid-naïve patients for clinical and PET/CT parameters. Further sub-analyses were conducted by the duration of steroid treatment (0 days, 1–3 days, 4–7 days, 8 days or more), average prednisone daily dose (0 mg, 1–20 mg, 21–59 mg, 60 mg or more), prednisone cumulative dose in the week prior to the PET/CT scan (0 mg, 1–140 mg, 141–479 mg, 480 mg or more) and prednisone mg per kilogram (kg) (< 0.5 mg/kg, 0.5 mg–0.99 mg/kg and ≥ 1 mg/kg).

### Primary outcome measure

The primary outcome measure was the effect of steroid intake on SUVmax in lesions of aggressive B cell lymphoma. The effect was examined across different steroid doses and regimens.

### Secondary outcome measures

The secondary outcome measures were the effect of steroid treatment on additional PET/CT parameters, including SUVmean, MTV, and tumor burden, in addition to SUVmax and SUVmean of the liver and mediastinum.

In addition, we examined whether disease and tumor characteristics influenced steroid administration prior to the PET/CT scan.

### Statistical analysis

The statistical analysis was generated using SAS, version 9.4. For the two groups (steroid-naïve and steroid-treated), continuous normally distributed parameters were analyzed using t-test, and continuous non-normally distributed parameters using Wilcoxon test.

When comparing multiple groups of unpaired samples, we used the Friedman (Ranked Anova) with Tukey–Kramer correction for multiple corrections in order to test for differences between groups.

Continuous parameters are presented as mean and standard deviation (SD) or median and interquartile ratio (IQR), as appropriate. Fisher’s exact test was used to compare the value of categorical variables between study groups. Categorical variables are presented as number and percentage. Two-sided *P* values less than 0.05 were considered statistically significant.

## Results

### Patients and disease characteristics

Of the 234 adult patients diagnosed with aggressive non-Hodgkin B cell lymphoma at our center during the study period, 178 had a baseline PET/CT scan that was available for review, and these patients were included in our analysis. Our cohort included 99 male (55.62%) and 79 female patients of median age 67 years (range 18–93). The specific diagnosis was diffuse large/high-grade B cell lymphoma in 160 patients (89.89%), primary mediastinal (thymic) large B cell lymphoma in 13 (7.31%), and Burkitt lymphoma in 5 (2.81%).

131 patients (73.5%) were steroid-naïve and 47 (26.4%) received steroids within 30 days before the PET/CT scan. The mean duration of steroid treatment was 10.15 ± 9.45 days. The average daily dose was equivalent to 69.55 ± 36.31 mg of prednisone, and the mean cumulative prednisone dose during the week prior to PET/CT scanning was equivalent to 353.43 ± 239.93 mg. The mean prednisone dose adjusted for weight was 1 ± 0.58 mg/kg per day.

Data on glucose levels prior to PET CT scan were available in 83 patients (46% of the entire cohort). Of these, 49 (59%) patients were in the steroid-naïve group and 34 (41%) were in the steroid treatment group. The mean glucose level in the steroid- naïve group was 115.4 ± 47.73 mg/dl and in the steroid-treated group 130.47 ± 54.60 mg/dl (*p* = 0.17).

There was no statistically significant difference between the steroid-treated and steroid-naïve groups in baseline characteristics of age, sex, diabetes mellitus and ki67 proliferation index. The steroid-treated group had a significantly higher disease stage and higher mean LDH level and had a trend toward a higher IPI score, compared to the steroid-naïve group (Table 1).

**Table 1:** Characteristics of patients with aggressive non-Hodgkin lymphoma by pre-PET-CT steroid treatment

Characteristics	Steroid-naïve (*n* = 131)	Steroid-treated (*n* = 47)	*P* value
Female sex, *n* (%)	55 (41.99%)	24 (51.1%)	0.31
Age (years), mean (SD)	64.63 (16.13)	63.79 (15.38)	0.75
Diabetes mellitus, *n* (%)	30 (22.9%)	12 (25.5%)	0.69
LDH level (U/L), mean (SD)	881.64 (1996.93)	2614.81 (11,762.75)	0.03
Ki67 index (%), mean (SD)	71.89 (19.68)	74.5 (20.02)	0.32
*IPI score, n (%)*			
0–1	44 (33.59)	12 (25.3)	
2–3	72 (54.96)	24 (51.07)	
4–5	15 (11.45)	11 (23.44)	0.12
Mean IPI score (SD)	2.10 (1.14)	2.51 (1.37)	0.07
*Stage, n (%)*			
1	26 (19.85)	2 (4.26)	
2	19 (14.51)	11 (23.41)	
3	17 (12.98)	3 (6.38)	
4	69 (52.67)	31 (65.95)	0.02
Mean stage, *n* (SD)	2.98 (1.22)	3.34 (0.98)	0.07

*LDH* lactate dehydrogenase, *IPI* international prognostic index

### Primary outcome: SUVmax

**Fig. 1:**
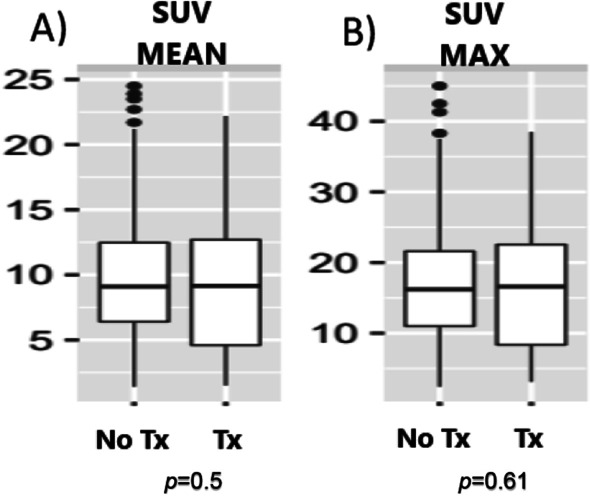
Box Plot of SUV in steroid naïve (no Tx) and steroid-treated (Tx) patients. **A** SUV mean, **B** SUV max. Tx—treatment

There was no statistically significant difference in SUVmax between the steroid-naïve group and either the whole steroid-treated group: median and IQR 16.2 (11,21.6) and 16.4 (10.2,21.9), respectively; *P* = 0.61 (Fig. 1).

There was no difference in SUVmax also in analysis of subgroups of steroid-treated patients divided by duration of steroid treatment, average daily dose of prednisone, cumulative dose of prednisone in the week prior to the PET/CT scan, or by weight-adjusted (mg/kg) prednisone dose, using the Friedman (Ranked Anova) test (Table 2). A post hoc analysis was not performed because of lack of significant difference in the aforementioned analysis.

**Table 2:** SUVmax across various prednisone dosage and durations

Characteristics of steroid treatment	No. patients	Median, (Q1,Q3)	*P* value (Friedman test)
Duration of steroid use before PET-CT, days			0.39
0	131	16.1 (10.9,21.6)	
1–3	12	20.7 (13.7,33.7)	
4–7	16	16.7 (5.5,22.6)	
8+	19	14.9 (8.4,22.15)	
Average daily prednisone dose, mg			0.96
0	131	16.1 (10.9,21.6)	
1–20	7	17.8 (8.3,23.6)	
21–59	4	21.9 (10.2,22.4)	
60+	36	16.5 (8.5,22.6)	
Cumulative prednisone dose in week before PET-CT, mg			0.94
0	131	16.1 (10.9,21.6)	
1–140	12	16.8 (10.2,24.7)	
141–479	22	15.2 (7.9,22.2)	
480+	13	18.2 (8.1,22.6)	
Average mg/kg daily prednisone dose			0.86
0	131	6.1 (10.9,21.6)1	
0.01–0.49	8	18.8 (8.3,23.6)	
0.5–0.99	14	15.6 (10.2,18.2)	
1+	25	18.5 (8.5,25)	

*PET-CT* positron emission tomography computed tomography

### Secondary outcomes: other PET/CT parameters

**Fig. 2:**
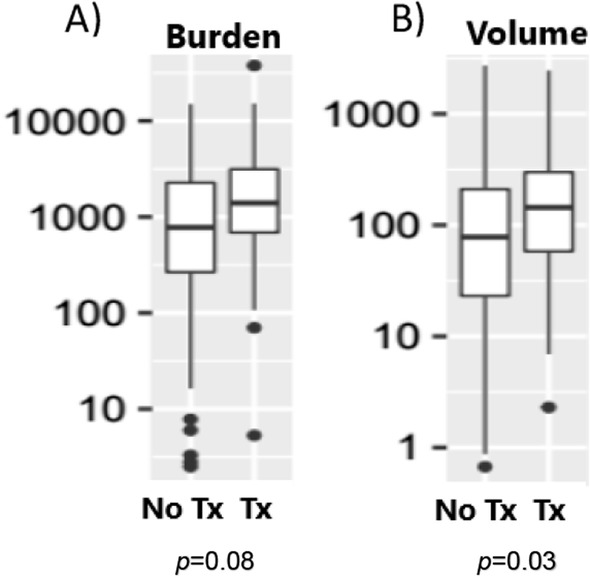
Box Plot of tumor PET CT characteristics in steroid naïve (no Tx) and steroid-treated (Tx) patients. The numbers of the Y axis are presented in Log scale, **A** tumor burden **B** tumor volume (cm^3^)

The steroid-treated patients had a significantly larger mean tumor volume compared to the steroid-naive patients with a median of 144 ( IQR 56.3,302.7) cm^3^ versus 78.10 (IQR 20.30,214) cm^3^, *P* = 0.03. They also had a trend toward a higher tumor metabolic burden (Fig. 2), although the difference did not reach statistical significance, median of 1400.4 (IQR 678.5,3185.6) versus 778.1 (IQR 262.7,2281.4), *p* = 0.08.

There were no statistically significant between-group differences in the other PET/CT parameters evaluated (SUVmean, liver and mediastinum SUVmax and SUVmean).

## Discussion

In the current study we observed that in patients with aggressive B cell non-Hodgkin’s lymphoma, there was no difference in mean SUVmax between those who were treated with steroids before the PET/CT scan and those who were not, regardless of the steroid dose or the duration of treatment. Most other PET/CT parameters were also not affected by steroids given prior to the imaging. These findings imply that pretreatment with steroids does not decrease the diagnostic yield or the staging accuracy of PET/CT in this patient population. This has important implications given the steroid-sensitive nature of lymphoma tumors, the important role of steroids in lymphoma therapy and the clinical need for early (pre-scanning) administration of steroids in some patients with severe symptoms or risk of organ damage.

Although recommendations for tumor imaging do not prohibit pre-scanning steroids [15], concern of a possible disruptive effect of steroids might cause treating oncologists to avoid their use in such circumstances. Our data sheds light on this common clinical dilemma.

Most of the limited data on the effect of steroid treatment on PET/ CT scan results in the literature has been derived from studies on patients with autoimmune/rheumatic diseases. Results to date have been conflicting, though some studies have shown a disruptive impact, suggesting a loss of PET/CT scan sensitivity, worsened with prolonged steroid treatment. In one study of patients with giant cell arteritis, seven to ten days of steroid treatment decreased PET/CT scan sensitivity, whereas treatment for three days or less did not [11, 16]. In the field of oncology, despite the widespread use of PET/CT scans, there is paucity of published data on the impact of pre-scanning steroids on PET/CT imaging. One preclinical study in a rat model of hepatocellular carcinoma demonstrated that pretreatment with steroids increased blood glucose levels, yet this did not significantly impact FDG uptake [17]. In a small prospective trial, 17 patients with non-small cell lung cancer (NSCLC) underwent PET/CT scan with or without pretreatment with 8 mg oral dexamethasone, administered 24 h prior to PET/CT followed by surgical resection of the tumor and lymph nodes. There was no significant difference in visual score between the two groups, although SUVmax was significantly lower in the dexamethasone-treated group. There was no between-group difference in the evaluation of true-positive nodes for malignancy. Nonetheless, visual score and SUVmax were significantly lower after dexamethasone pretreatment in the false-positive nodes. The authors concluded that dexamethasone treatment improved PET/CT scan accuracy in patients with NSCLC and helped identify the involved lymph nodes [18].

One of the few studies regarding the effect of pre-scanning steroid treatment on PET/CT results in patients with lymphoma, included 130 patients with central nervous system lymphoma. Steroid treatment for more than one week was associated with a higher rate of negative brain PET/CT scans compared to shorter duration of treatment. In the patients with a positive brain PET/CT scan, steroids did not impact SUVmax [19].

In the present study, steroid treatment was not associated with a change in SUVmax measurements across various steroid dosages and durations. This was also true for most other PET/CT parameters examined. Considering that we focused specifically on aggressive B cell lymphomas, mostly DLBCL, it is possible that the tumor sensitivity to steroids was outweighed by the extremely aggressive nature of the disease.

Tumor burden parameters at baseline were higher in the steroid-treated group, most probably reflecting the reason for the early initiation of steroid treatment. Accordingly, we observed that patients who received steroids had higher LDH levels, IPI scores, and Lugano stage, in line with the higher tumor load and likely greater disease clinical severity at presentation, which led to the clinical need for early steroid treatment. Indeed, despite the precedent steroid treatment, the tumor volume and tumor metabolic burden were higher compared to the steroid-naïve patients.

Approximately half of the patients in our cohort had available glucose levels prior to their baseline PET CT. Although all patients were treated at our institution and all imaging studies were reviewed by our nuclear medicine department, the scans took place at various institutions. Each facility has its own policy regarding the need for glucose level testing prior to PET CT examination, and some facilities do not routinely perform glucose level testing prior to PET CT scans, unless the patients have diabetes or feel unwell. This practice is based on several published reports that have demonstrated that the impact of glucose levels on SUVs in most organs is negligible, and delaying the scan might harm the patient [20–23]. Nonetheless, among the patients for whom glucose data were available, there was no difference in the mean glucose levels between the steroid-treated and steroid-naïve groups.

Our study was limited by the retrospective design, with its inherent biases. Nonetheless, the key demographic data were comparable between the steroid-naïve and steroid-treated groups. A prospective trial design could potentially provide more conclusive results, yet the methodological difficulties in designing such a trial would be challenging. Despite the retrospective nature of the present study, we were able to demonstrate, in a relatively large cohort of aggressive B cell lymphoma patients, that in the real-life setting steroid-treated patients had similar key PET parameters compared to those who were steroid-naïve. Another limitation is that the steroid-treated group accounted for only one-third of the entire cohort, and the dose and duration of steroid treatment varied. These factors may have hampered the subgroup analyses.

Bearing in mind the above mentioned limitations, our study did not show a decrease in the diagnostic yield of baseline PET/CT scans in patients with aggressive B cell lymphoma who received steroids prior to the scan, despite lymphoma being a steroid-sensitive disease. Physicians considering prescribing steroids prior to baseline PET/CT in patients with aggressive B cell lymphoma, particularly in the presence of impending organ damage or significant disease symptoms, might be concerned that the steroids might reduce the ability of PET CT to properly demonstrate the malignancy. Our findings shed some light on this common clinical dilemma that has thus far had scarce literature to support any informed decision.

In conclusion, to the best of our knowledge, this is the first study to assess the effect of steroid treatment on the interpretation of the baseline PET/CT scan in patients with aggressive B cell lymphoma. We found no difference in SUVmax and most other PET/CT parameters between patients who were or were not treated with steroids prior to PET/CT. Since this is a retrospective study, and several differences in disease characteristics were evident between the steroid-naïve and steroid-treated groups, our results should be interpreted with caution. Further studies are needed to fully assess the effect of steroids on PET/CT results in patients with other types of hematological and solid malignancies.

## Data Availability

The data that support the findings of this study are available from the corresponding author upon reasonable request.
